# Genetic justification of COVID‐19 patient outcomes using DERGA, a novel data ensemble refinement greedy algorithm

**DOI:** 10.1111/jcmm.18105

**Published:** 2024-02-09

**Authors:** Panagiotis G. Asteris, Amir H. Gandomi, Danial J. Armaghani, Markos Z. Tsoukalas, Eleni Gavriilaki, Gloria Gerber, Gerasimos Konstantakatos, Athanasia D. Skentou, Leonidas Triantafyllidis, Nikolaos Kotsiou, Evan Braunstein, Hang Chen, Robert Brodsky, Tasoula Touloumenidou, Ioanna Sakellari, Nizar Faisal Alkayem, Abidhan Bardhan, Maosen Cao, Liborio Cavaleri, Antonio Formisano, Deniz Guney, Mahdi Hasanipanah, Manoj Khandelwal, Ahmed Salih Mohammed, Pijush Samui, Jian Zhou, Evangelos Terpos, Meletios A. Dimopoulos

**Affiliations:** ^1^ Computational Mechanics Laboratory, School of Pedagogical and Technological Education Athens Greece; ^2^ Faculty of Engineering & IT University of Technology Sydney Sydney New South Wales Australia; ^3^ University Research and Innovation Center (EKIK), Óbuda University Budapest Hungary; ^4^ School of Civil and Environmental Engineering University of Technology Sydney Sydney New South Wales Australia; ^5^ 2nd Propedeutic Department of Internal Medicine Aristotle University of Thessaloniki Thessaloniki Greece; ^6^ Hematology Division Johns Hopkins University Baltimore USA; ^7^ Hematology Department – BMT Unit G Papanicolaou Hospital Thessaloniki Greece; ^8^ College of Civil and Transportation Engineering Hohai University Nanjing China; ^9^ Civil Engineering Department National Institute of Technology Patna Patna India; ^10^ Department of Engineering Mechanics Hohai University Nanjing China; ^11^ Department of Civil, Environmental, Aerospace and Materials Engineering University of Palermo Palermo Italy; ^12^ Department of Structures for Engineering and Architecture University of Naples “Federico II” Naples Italy; ^13^ Engineering Faculty San Diego State University San Diego California USA; ^14^ Department of Geotechnics and Transportation, Faculty of Civil Engineering Universiti Teknologi Malaysia Johor Bahru Malaysia; ^15^ Institute of Innovation, Science and Sustainability Federation University Australia Ballarat Victoria Australia; ^16^ Engineering Department American University of Iraq Sulaymaniyah Iraq; ^17^ School of Resources and Safety Engineering Central South University Changsha China; ^18^ Department of Clinical Therapeutics, Medical School, Faculty of Medicine National Kapodistrian University of Athens Athens Greece

**Keywords:** artificial intelligence, classification algorithms, COVID‐19, DERGA, genetic, SARS‐CoV2, variants

## Abstract

Complement inhibition has shown promise in various disorders, including COVID‐19. A prediction tool including complement genetic variants is vital. This study aims to identify crucial complement‐related variants and determine an optimal pattern for accurate disease outcome prediction. Genetic data from 204 COVID‐19 patients hospitalized between April 2020 and April 2021 at three referral centres were analysed using an artificial intelligence‐based algorithm to predict disease outcome (ICU vs. non‐ICU admission). A recently introduced alpha‐index identified the 30 most predictive genetic variants. DERGA algorithm, which employs multiple classification algorithms, determined the optimal pattern of these key variants, resulting in 97% accuracy for predicting disease outcome. Individual variations ranged from 40 to 161 variants per patient, with 977 total variants detected. This study demonstrates the utility of alpha‐index in ranking a substantial number of genetic variants. This approach enables the implementation of well‐established classification algorithms that effectively determine the relevance of genetic variants in predicting outcomes with high accuracy.

## 1 INTRODUCTION

The ongoing coronavirus disease (COVID‐19) pandemic, caused by the severe acute respiratory syndrome coronavirus‐2 (SARS‐CoV‐2), has resulted in remarkable global morbidity and mortality among patients.^1^ Despite continuing vaccination efforts, there is still a need to reduce the impact of the disease, particularly in specific populations. Studies have revealed that SARS‐CoV‐2 triggers a cycle of immune dysfunction, endothelial injury,^2^ and microangiopathy,^3^ resulting in severe COVID‐19 being characterized as a multisystemic vascular disease.^4^ Given that complement is a significant regulator of endothelial injury syndromes such as thrombotic microangiopathies (TMAs), and severe COVID‐19 seems to resemble complement‐mediated TMAs, researchers have studied the role of complement activation in severe COVID‐19^5^, ^6^ and discovered genetic variants that may increase an individuals' susceptibility to severe disease. Additionally, a number of studies have investigated the use of complement inhibitors as a potential treatment for severe COVID‐19,^7^, ^8^ with encouraging results mostly seen in case series. Complement inhibitors such as eculizumab,^9^, ^10^, ^11^, ^12^, ^13^, ^14^, ^15^ ravulizumab,^16^, ^17^ Cp40,^18^, ^19^ AMY‐101,^20^, ^21^ emapalumab,^22^ narsoplimab,^23^, ^24^ conestat alpha,^25^, ^26^ and LFG‐316^27^ have the potential to impact the treatment of severe disease. However, broader use of these drugs is limited by cost and accessibility, as well as the need for more appropriate patient selection and larger studies. To address these challenges, robust prediction tools utilizing critical genetic variants, age and gender are essential in identifying patients who may benefit from complement inhibition. The authors of this study aim to identify key complement‐related genetic variants that predict severe COVID‐19 using a recently proposed alpha‐index. This index was initially introduced for ranking haematological indices that impact the outcome of COVID‐19 cases.^28^ In addition, a novel data ensemble refinement greedy algorithm (DERGA) is utilized, in order to demonstrate the optimal subset combination (pattern) of these genetic variants with the best prediction accuracy regarding the outcome of each patient's illness.

## 2 MATERIALS AND METHODS

### 2.1 Study population

**FIGURE 1:**
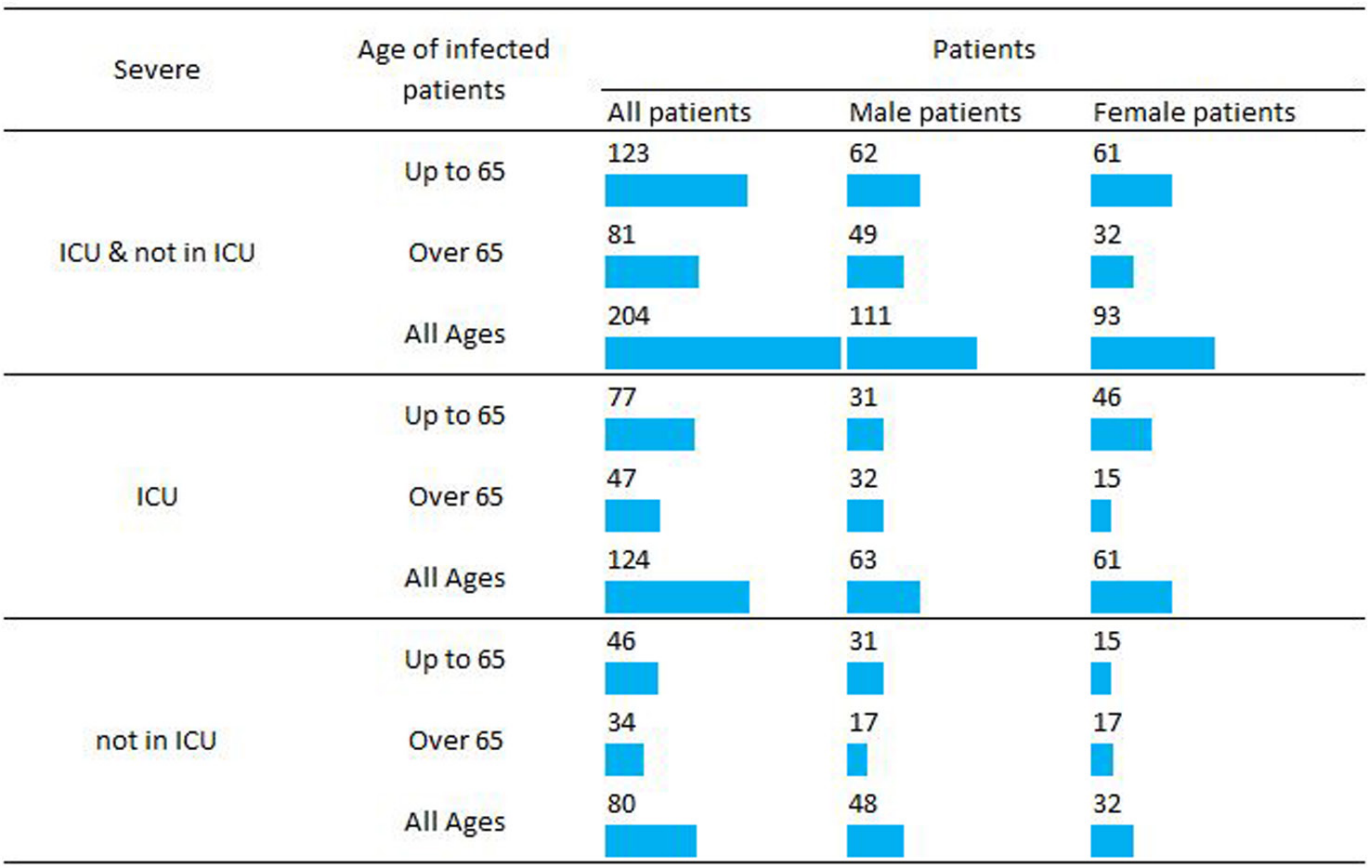
Study population categorized by age, gender and disease severity (requiring or not hospitalization in intensive care unit [ICU]).

Our study recruited adult patients who were hospitalized for COVID‐19 at three referral centres (Georgios Papanicolaou, Attikon Hospital and Johns Hopkins Hospital) from April 2020 to April 2021. We studied 204 patients, 124 hospitalized in intensive care units (ICU) and 80 in COVID‐19 general ward. Figure 1 summarizes demographics according to disease severity, age and gender. Participants were confirmed to have SARS‐CoV‐2 infection through RT‐PCR (reverse‐trancriptase polymerase chain reaction) testing. The medical history and progress of each patient were recorded by their treating physicians and followed until their discharge or death. Patients with non‐available data on clinical course and outcome were not included in the latter analysis. The study was approved by the Institutional Review Boards of the referral centres and conducted in accordance with the Declaration of Helsinki.

### 2.2 Genetic analysis

The study utilized next‐generation sequencing (NGS, Illumina, San Diego, California) to analyse DNA that was extracted from peripheral blood samples. The analysis focused on a panel of complement‐related genes, which included *complement factor H/CFH*, *CFH‐related*, *CFI*, *CFB*, *CFD*, *C3*, *CD55*, *C5*, *CD46* and *thrombomodulin/THBD*, as well as TMA‐associated *ADAMTS13* (a disintegrin and metalloproteinase with thrombospondin type 1 motifs). The design of probes was done using DesignStudio (Illumina, San Diego, California) to include all exons and an additional 15 bases of the intronic regions, resulting in 98% coverage. The initial amount of DNA material used was 10 ng per pool and the libraries were quantified using Qubit (Thermofisher Scientific, Waltham, Massachusetts). The sequencing of the libraries was performed on an Illumina System in a 2 × 150 bp run (Illumina, San Diego, California). Both Ensembl and Refseq resources were utilized to annotate the output files. The variants' clinical significance was determined using ClinVar and the current version of the Complement Database.

### 2.3 Compilation of genetic variants database

According to the genetic analysis conducted in 204 patients with COVID‐19, a database, that was comprised from 204 datasets and corresponded to the 204 patients, was synthesized. Each dataset was specified by 980 parameters. The first two parameters corresponded to age and gender of the patient, the next 977 corresponded to genetic variants detected in the patient, and the last parameter specified the severity of the patient's illness. Database is appended to this paper as Data S2 (excel file entitled Database—with all 977 Genetic Variants).

### 2.4 Optimal pattern of variants affecting the COVID‐19 outcome

The main objective of this study is to identify the optimal pattern of genetic variants that determines the outcome of the patients' illness, specifically whether they require admission to the intensive care unit (ICU). To achieve this goal, appropriate techniques and algorithms of artificial intelligence have been employed, under the assumption that the number of variants and their respective possible combinations were not excessively large. Additionally, the database must be reliable and able to statistically describe the phenomenon being studied.

The database for 204 patients and 977 variants has been analysed and the possible combinations have been determined through the application of the following equation.

(1) $$\text{Combinations}=2\sum_{i=1}^{\mathrm{nv}}\frac{\mathrm{nv}!}{i!\left(\mathrm{nv}-i\right)!}=2\left(2^{\mathrm{nv}}-1\right)$$

where *nv* is the number of the genetic variants in database. Setting the value *nv* = 977, we get 2.554676 × 10^294^ possible combinations.

To address this issue, two objectives were identified: (i) reduce the number of 977 variants to a subset which contains the most crucial variants that predict the outcome of the patients' illness and can be rapidly computed, and (ii) identify the optimal pattern using only this subset of crucial variants.

Taking into consideration these objectives, the next two sections present a recently proposed index for identifying the most crucial variants and a novel algorithm for identifying the optimal subset of variant combinations.

### 2.5 Crucial genetic variants

In order to reduce the 977 variants into a much smaller subset which comprise only the variants that affect outcome of the disease, alpha‐index, which was recently proposed by the authors for ranking haematological indices that also affect the outcome of patients with COVID‐19, was utilized.^28^ This index is defined as

(2) $$\text{alpha}\;\left(i\right)-\text{index}=\left(\mu_{i}^{\text{notinICU}}-\mu_{i}^{\text{inICU}}\right)100$$

where

*i* corresponds to *i*_th_ genetic variant (*i* = 1–977),

$\mu_{i}^{\text{not in}\;\mathrm{ICU}}$ is the mean value of the *i*_th_ genetic variant for COVID‐19 infected patients who did not require hospitalization in ICU and$\;\mu_{i}^{\text{in}\;\mathrm{ICU}}$ is the mean value of the *i*_th_ genetic variant for COVID‐19 infected patients who require hospitalization in ICU.

Based on the above equation, the index takes values between −100 and 100. A genetic variant's effectiveness in determining whether a patient will be admitted to the ICU is directly proportional to this index value:

1. First, if a genetic variant is present in both sets of patients (ICU/not in ICU) the index has a value of 0, indicating that the variant does not affect a patient's admittance to the ICU.
2. Second, if a genetic variant is present only in the set of patients admitted to the ICU and not present in the set of patients not admitted to the ICU the index has a value of −100, indicating that the variant has a significant effect on a patient's admittance to the ICU.
3. Third, if a genetic variant is not present in the set of patients admitted to the ICU and is present only in the set of patients not admitted to the ICU the index has a value of 100, indicating that the variant has a significant effect on a patient's non‐admittance to the ICU.

The above‐stated index was used to rank the 977 variants. A subset of the most crucial variants that segregate with requirement for hospitalization in the ICU was selected.

### 2.6 DERGA, the proposed greedy algorithm

Based on the previously presented alpha‐index, the number of genetic variants that predict severity of a COVID‐19 patient's illness can be significantly reduced. However, the number of possible combinations of these variants that can be used as input parameters in a classification model to predict ICU admission remains large, and the solution process remains challenging. To address this issue, a new data ensemble refinement greedy algorithm (DERGA) is proposed in this section. The objective of DERGA is to identify the optimal combination of essential genetic variants by first ranking them through the alpha‐index and subsequently employing a set of classification algorithms with combinations of the remaining variants, after ranking them using the alpha‐index.

The proposed algorithm can be described in the following finite number of steps:

Step 1. A set Α, |Α| = *m*, is defined using widely adopted classification algorithms in the literature. Each algorithm, A_*i*_, *i* = 1, …, *m*, will be fitted to training data for predicting if a COVID‐19 patient will be admitted to ICU or not.

Step 2. During the training and development of the heuristic classification algorithm A_i_ in Step 1, the entirety of the genetic variants (nm) that have been selected using the alpha‐index are used as input parameters. Performance indices are determined with respect to the achieved prediction level.

Step 3. Next, the algorithm is fitted for nm cases of parameters, with one genetic variant removed each time. Performance indices are determined for each case, with respect to the achieved prediction level. From the nm cases of algorithm execution, the one that corresponds to the smallest value of the performance index for the prediction level, defines which genetic variant affects the prediction level the least and is removed from the set of nm variants. This process is repeated for nm‐1 times, removing a variant each time.

With the completion of this procedure, the following are determined:

1. The achieved prediction for the case, where all genetic variants are used as input parameters in the currently executed heuristic classification algorithm.
2. The optimal combination of genetic variants (pattern) in the currently executed heuristic classification algorithm that corresponds to the best prediction level.
3. The ranking of all variants according to their contribution to the prediction.
4. The ranking of the remaining variants according to their significance of prediction in contrast to other black box metaheuristics, which only determine the remaining variants and not their relative significance.
5. The most crucial variant, which is the one remaining during the repetitive process of the proposed algorithm.
6. The above five findings correspond to each executed algorithm *A*_*i*_. The optimal among all the algorithms executed define the best algorithm and the global optimum genetic variant pattern, as well as the global crucial genetic variant.

The proposed algorithm (DERGA), is characterized as greedy and local hill‐climbing heuristic, as it seeks to remove the variant (input parameter) that contributed the least to the prediction level in each iteration *j* = 0,…,29 of the currently executed algorithm A_*i*_, *i* = 1,2,…,*m*. By removing a variant from the training datasets in each iteration, the algorithm makes data reduction in steps of removing columns from the training data.

The reliability of the proposed algorithm is established by the magnitude of the achieved prediction level. The greater the prediction score, the greater the reliability of the proposed algorithm and the proposed combination of genetic variants (patterns). Additionally, for the studied case of predicting if a COVID‐19 patient will require hospitalization in ICU or not, the achieved accuracy of prediction must be greater than 95%.

## 3 RESULTS

**FIGURE 2:**
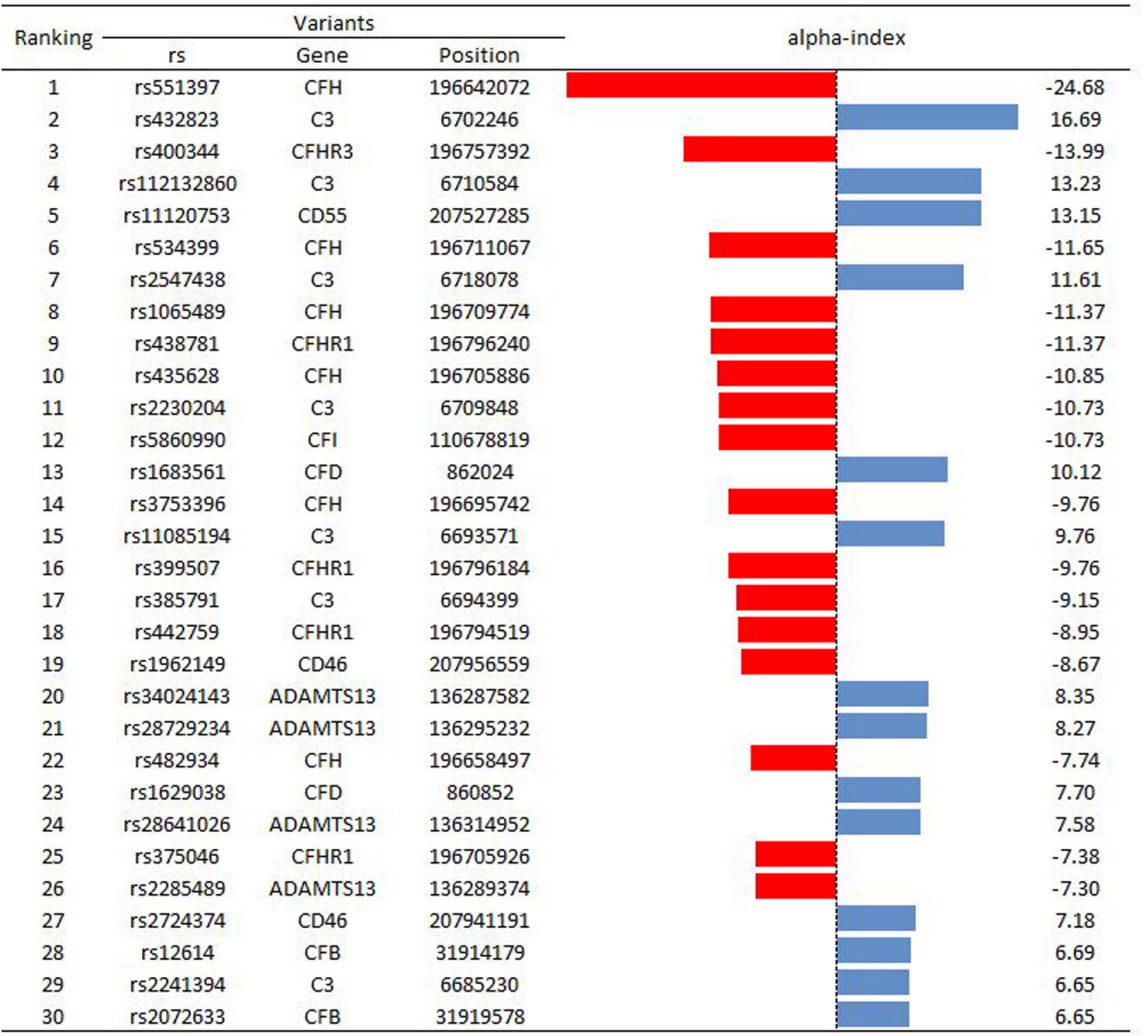
Ranking of the top 30 genetic variants based on the proposed new alpha‐index. Red colour signifies that the occurrence of the variant is dominating in patients admitted to ICU, while blue signifies the occurrence of the variant for those not admitted in ICU.

The proposed algorithm used a database of 204 COVID‐19 patients, consisting of 204 datasets and containing 977 genetic variants. The number of genetic variants per patient varied ranging from 40 to 161. By applying the alpha‐index, the 30 most crucial genetic variants were identified and ranked in decreasing absolute value, as shown in Figure 2. The database of 30 most crucial genetic variants is appended to this paper as Data S1 (excel file entitled Database—with 30 most crucial Genetic Variants).

The proposed DERGA algorithm was used to find the optimal combination of the 30 most crucial genetic variants, by utilizing five different classification algorithms. These algorithms were selected from widely adopted and available literature, including Decision Trees,^29^ Extra Trees,^30^ Random Forrest,^31^ Gradient Boost^32^ and Gaussian Process classification algorithms,^33^ for their superior performance in solving the current problem.

The 204 datasets of the database, containing the 30 most crucial genetic variants, were divided into two distinct groups. Specifically, one group, constituting 70% of the data and referred to as the Training datasets, was utilized for the training of the proposed algorithm. The other group consisted of the remaining 30% of the data, termed the Testing datasets, employed to assess the performance of the algorithm. Notably, these two data groups were selected from 10 random partitions (70–30) to minimize performance indices deviation between Training and Testing datasets. This careful selection enhances the reliability and robustness of the algorithm evaluation process.

**FIGURE 3:**
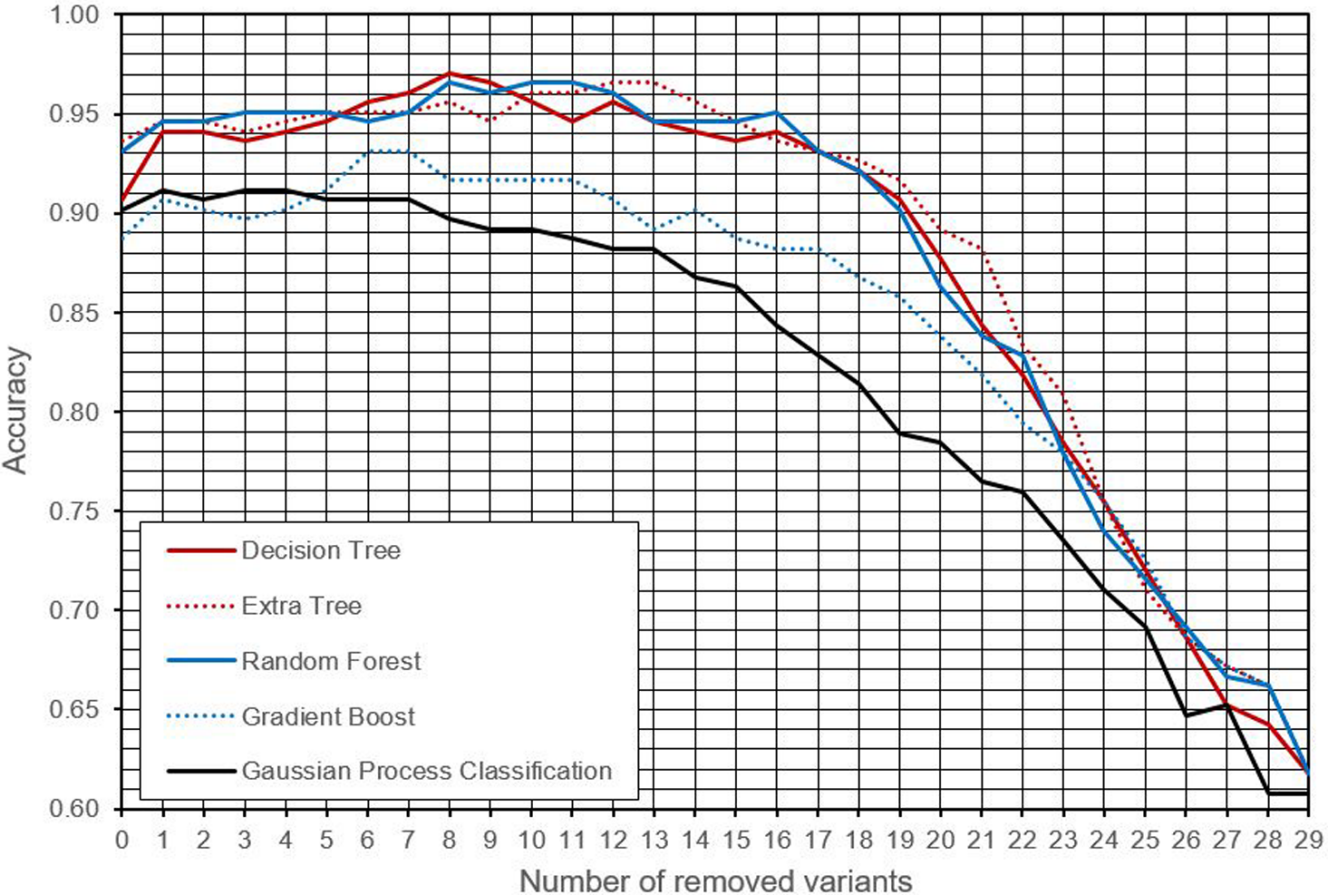
Accuracy plots for the DERGA procedure for each used classification algorithm.

Accuracy plots of proposed DERGA algorithm for the five different classification algorithms are demonstrated in Figure 3. Additionally, Table 1 presents the achieved performance indices^34^, ^35^, ^36^, ^37^ for each of these five algorithms, along with the number of genetic variants used as input parameters for the best prediction score of whether a COVID‐19 patient was admitted to the ICU or not.

**TABLE 1:** Performance indices of the five executed under DERGA classification algorithms.

Ranking	Algorithm	Number of included genetic variants (input parameters)	Performance Indices					
			Accuracy	Precision	F1‐Score	Recall	Specificity	Sensitivity
1	Decision Trees	22	0.9706	0.9625	0.9625	0.9625	0.9625	0.9758
2	Extra Trees Classification	18	0.9657	0.9740	0.9554	0.9375	0.9375	0.9839
3	Random Forrest	20	0.9657	0.9620	0.9560	0.9500	0.9500	0.9758
4	Gradient Boost	24	0.9314	0.9459	0.9091	0.8750	0.8750	0.9677
5	Gaussian Process Classification	29	0.9118	0.9189	0.8831	0.8500	0.8500	0.9516

Figure 3 demonstrates the efficacy of proposed algorithm in successively identifying and eliminating the least critical genetic variants from the initial set of 30 key variants selected with the alpha‐index (Figure 1). The peak of the curve for each algorithm represents the maximum prediction score attainable with that particular algorithm and determines the number of parameters, that is, genetic variants that are omitted and not considered in the estimation process for determining ICU admission for a patient.

The results in Figure 3 and Table 1 display that the Decision Trees algorithm performed best, with an accuracy of 0.9706, while only employing 22 out of the 30 genetic variants. Table 2 lists the genetic variants that were used as input parameters for the optimal Decision Tree classifier. The ranking in the leftmost column is based on the reverse order of removal of variants during the execution of the proposed DERGA algorithm.

**TABLE 2:** Ranking of genetic variants used as input parameters in proposed optimal DERGA‐Decision Tree algorithm.

Ranking		Variants		
DERGA	Alpha‐index	rs	Gene	Position
1	1	rs551397	CFH	196,642,072
2	11	rs2230204	C3	6709,848
3	28	rs12614	CFB	31914,179
4	12	rs5860990	CFI	110,678,819
5	23	rs1629038	CFD	860,852
6	19	rs1962149	CD46	207,956,559
7	7	rs2547438	C3	6718,078
8	26	rs2285489	ADAMTS13	136,289,374
9	2	rs432823	C3	6702,246
10	24	rs28641026	ADAMTS13	136,314,952
11	9	rs438781	CFHR1	196,796,240
12	29	rs2241394	C3	6685,230
13	8	rs1065489	CFH	196,709,774
14	10	rs435628	CFH	196,705,886
15	3	rs400344	CFHR3	196,757,392
16	17	rs385791	C3	6694,399
17	5	rs11120753	CD55	207,527,285
18	16	rs399507	CFHR1	196,796,184
19	14	rs3753396	CFH	196,695,742
20	22	rs482934	CFH	196,658,497
21	4	rs112132860	C3	6710,584
22	6	rs534399	CFH	196,711,067

The high prediction score achieved demonstrates the effectiveness and reliability of the proposed DERGA algorithm. Additionally, it is noteworthy that for the optimal Decision Trees algorithm as well as for all the algorithms studied, the most crucial variant is rs551397 (gene *CFH*), which confirms the reliability of the alpha‐index for ranking genetic variants in terms of their association with ICU admission.

## 4 DISCUSSION

In this study, we introduce a novel prediction tool based on robust variables which demonstrates a high degree of accuracy in predicting the outcome of COVID‐19. Additionally, this study showcases the reliability of the recently proposed alpha‐index^28^ in ranking genetic variants according to their impact on disease outcomes.

To date, genome‐wide association studies (GWAS) have identified multiple genetic loci that are either associated with intense disease severity or increased susceptibility to COVID‐19.^38^ For disease severity, key findings include variants in genes such as *DPP9*,^39^ *TLR7*,^40^, ^41^ *IFNAR2* and *FOXP4*.^14^ In addition, associations have been observed with genes that modulate the immune response to viral infection, such as *TYK2* ^39^ and *IFNAR2*.^14^, ^38^ Regarding COVID‐19, genetic susceptibility is primarily linked to polymorphisms in the angiotensin‐converting enzyme 2 (*ACE2*) gene,^14^, ^42^, ^43^ ABO blood group,^44^, ^45^ *SLC6A20* gene^46^, ^47^ and interferons.^43^, ^48^

As far as complement‐related variants, few studies about COVID‐19 have emerged with significant outcomes. A recent study, aimed at exploring the association between genetic variation at chromosome 3p21.31 and the ABO blood group with complement activation and COVID‐19 severity, identified a variant (rs11385942) that predisposes individuals to severe COVID‐19. This variant was found to be associated with increased complement activation, as evidenced by elevated levels of circulating *C5a*, *sC5‐C9 and C5a* in individuals belonging to the non‐O blood group.^49^ Moreover, a genetic and transcriptional analysis documented 23 study‐wide significant SNPs in 12 complement genes.^50^ Integrative analysis of these data highlighted 4 SNPs in human complement genes (*C4BPA*, *C5AR1 and C3*) that encode for missense polymorphic variants (rs2230199, rs1047286, rs45574833 and rs4467185) associated with SARS‐CoV‐2 susceptibility.^51^ In addition, Delanghe et al characterized *C3* polymorphisms as confounders in the spread and outcome of COVID‐19 using a multivariate model.^52^

There are limited tools for prediction of COVID‐19 disease severity that can be applied to clinical practice or trials. We recently developed an algorithm to identify variants in *C3*, *CFH and THBD* that predict COVID‐19 severity.^36^ The algorithm predicted COVID‐19‐related ICU hospitalization based on a combination of variants with a rate of over 80%; however, it did not account for key morbidity and mortality factors, such as age and gender. To overcome this limitation, we improved the algorithm to include both ICU and non‐ICU patients and identified variants in complement‐related genes (*CFRH*, *THBD*, *C3 and CFH*), known to be dysregulated in complement‐related disorders.^35^ The updated algorithm was further implemented using an Artificial Neural Network (ANN) that incorporated age and gender, providing not only the ability to predict morbidity but also mortality in COVID‐19 patients. The present study expands upon our prior work through the use of the recently proposed alpha‐index^28^ to identify critical complement‐related genetic variants. These variants, when combined with the application of a novel data ensemble refinement procedure (DERGA algorithm) based on six different classification algorithms, yielded a remarkable predictive score for the ICU admission of COVID‐19 patients. For instance, DERGA‐Decision Tree algorithm managed to attain a 97% prediction accuracy using only 22 key variants, a result that has not been achieved in previous works.

Gender is considered a major risk factor for COVID‐19 disease. Healthy male individuals show higher levels of complement activation and increased morbidity and mortality.^53^, ^54^

Studies to date support an important role for the alternative pathway of the complement system in COVID‐19 pathogenesis, as it is directly activated by SARS‐CoV‐2.^55^ Based on the results of alpha‐index ranking, as well as the removal turn of each classification algorithm, the most crucial genetic variant was rs551397, which has been characterized as a high‐risk factor for age‐related macular degeneration (AMD).^56^ In accordance with our findings, recent studies have demonstrated that COVID‐19 patients with AMD are at a significantly increased risk of experiencing severe disease and death.^50^ The combination of genetic variants in complement‐related genes identified in our study may be suggestive of COVID‐19 disease biology.

The utilization of machine learning techniques has been employed in the development of prediction models for COVID‐19. These models have incorporated various data sources, including comorbid diseases,^57^, ^58^ clinical factors,^59^, ^60^ genetic factors^39^, ^42^, ^61^ and SARS‐COV‐2 viral clades.^62^, ^63^, ^64^ Given the promising results obtained from therapeutic approaches, including complement inhibition,^65^ in the treatment of COVID‐19, the development of reliable prediction tools based on complement‐related variants is of utmost importance. The utilization of similar tools in the precision medicine era, holds the potential for early patient identification and the implementation of a personalized, secure and effective therapeutic approach.^66^

## 5 LIMITATIONS AND FUTURE WORK

The major limitation of this study is the moderate number of patients comprising the variants database. The authors intend to increase the size of the database by collecting data from various sources in future work. This will lead to greater reliability of the classification procedures presented in this work and establish them as a valuable tool for predicting admittance to ICU for COVID‐19 patients. Additional limitations include the inability of our model to account for the effect of vaccination status on clinical outcome, as many of our samples were collected prior to the availability of widespread vaccination. Further, the majority of patients in our study were infected with the alpha variants of SARS‐CoV‐2 and therefore, the effects of the individual spike protein variants on disease severity are not extensively studied in our model. Moreover, our cohort comprised only from adult patients. In the paediatric population, the identification of novel complement variants^67^ poses a challenge to the generalization of our findings. Consequently, there is a need for additional studies to address this limitation. Lastly, our model provides high accuracy and prediction rates irrespectively of traditional confounders and comorbidities.

## 6 CONCLUSIONS

This study shows the effectiveness of using the recently proposed alpha‐index to rank a large number of genetic variants. This facilitates the use of well‐established classification algorithms in the machine learning literature, which are orchestrated in a data ensemble refinement procedure. The procedure is used to quickly and effectively determine the significance and relevance of the genetic variants in predicting the admittance of COVID‐19 patients in the ICU, with a high accuracy.

Studies have indicated the existence of genetic polymorphisms, in genes responsible for encoding complement proteins across diverse populations.^68^ Such genetic variations have been associated with disparities in complement function and regulation. The implications of these genetic differences extend to influencing susceptibility to specific diseases and responses to infections. Consequently, there is a pressing need for further research endeavours to deepen our understanding of this complex interplay.

Given the evolving landscape of literature on the long‐term implications of COVID‐19,^69^ in order to attain risk prediction within comparable accuracy and sensitivity, further large and high‐quality studies are needed.

In summary, it is worth noting that the innovative DERGA algorithm proposed in this study can be applied to a broad spectrum of classification problems. This versatility extends to various domains, including the medical field, where it can contribute to unveiling the nature of cardiovascular diseases, as well as in engineering and scientific applications. Particularly in scenarios with a substantial number of parameters, the suggested DERGA algorithm has the potential to prove highly effective. The demonstrated versatility positions it as a promising and effective tool with potential applications across diverse fields.

## FUNDING INFORMATION

This research did not receive any specific grant from funding agencies in the public, commercial or not‐for‐profit sectors.

## AUTHOR CONTRIBUTIONS

**Panagiotis G. Asteris:** Conceptualization (equal); methodology (equal); software (equal); supervision (equal); writing – original draft (equal). **Amir H. Gandomi:** Methodology (equal); software (equal); writing – original draft (equal). **Danial J. Armaghani:** Methodology (equal); software (equal); writing – original draft (equal). **Markos Z. Tsoukalas:** Methodology (equal); software (equal); writing – original draft (equal). **Eleni Gavriilaki:** Conceptualization (equal); validation (equal); writing – original draft (equal). **Gloria Gerber:** Methodology (equal); writing – review and editing (equal). **Gerasimos Konstantakatos:** Data curation (equal). **Athanasia D. Skentou:** Data curation (equal). **Leonidas Triantafyllidis:** Data curation (equal). **Nikolaos Kotsiou:** Writing – original draft (equal). **Evan Braunstein:** Methodology (equal). **Hang Chen:** Methodology (equal). **Robert Brodsky:** Methodology (equal). **Tasoula Touloumenidou:** Methodology (equal). **Ioanna Sakellari:** Methodology (equal). **Nizar Faisal Alkayem:** Methodology (equal). **Abidhan Bardhan:** Methodology (equal). **Maosen Cao:** Methodology (equal). **Liborio Cavaleri:** Methodology (equal). **Antonio Formisano:** Methodology (equal). **Deniz Guney:** Methodology (equal). **Mahdi Hasanipanah:** Methodology (equal). **Manoj Khandelwal:** Methodology (equal). **Ahmed Salih Mohammed:** Methodology (equal). **Pijush Samui:** Methodology (equal). **Jian Zhou:** Methodology (equal). **Evangelos Terpos:** Writing – review and editing (equal). **Meletios Dimopoulos:** Writing – review and editing (equal).

## CONFLICT OF INTEREST STATEMENT

Gloria Gerber received honoraria from Apellis Pharmaceutical. Gloria Gerber employment (spouse) and stock holder (spouse) at Pfizer. Eleni Gavriilaki is supported by the ASH Global Research Award and has consulted for Omeros Cooperation. The rest of authors do not have any conflicts of interest to disclose.

## CONSENT STATEMENT

Informed consent has been obtained from the patients.

## Supporting information


Data S1.

Data S2.

## Data Availability

The data that support the findings of this study are available from the corresponding author upon reasonable request.
